# Transmission Disequilibrium Test for quantitative traits based on multiple sibs

**DOI:** 10.1186/1755-8166-7-S1-P103

**Published:** 2014-01-21

**Authors:** Hemant S Kulkarni, Saurabh Ghosh

**Affiliations:** 1 Human Genetics Unit, Indian Statistical Institute, Kolkata, India

## Background

The Transmission Disequilibrium Test (TDT) is a well established family-based test for genetic association. The advantage of TDT over population-based association studies test, which is protected against population stratification, and hence, an association finding can be attributed to the presence of linkage. Quantitative traits (QT) are more informative on within-genotype variability and hence, statistical tests for identifying genes based on such traits tend to be more powerful compared to binary or qualitative traits. The TDT is based on a trio design (two parents and one offspring per family). However, if multiple sibs are present, TDT is only a valid test for linkage. In our study, we have extended the test for quantitative traits based on families with multiple offspring using a logistic regression framework.

## Materials and methods

We selected one offspring at random from each family to compute the usual TDT statistic. We then repeated the process and carry out separate permutation tests based on the mean and the maximum values of the TDT statistics obtained over different replications. We performed extensive simulations to evaluate the power of our proposed tests under a wide spectrum of genetic parameters (allele frequencies, QTL means and variances, extent of linkage disequilibrium between the QTL and the marker locus) and probability models (normal and chi-squares). We also compared the performance of the tests with a correction strategy using the Benjamini-Hochberg threshold.

## Results

We found that the test based on the mean TDT value is more powerful than that based on the maximum value. However, in either case, the power is higher compared to that obtained using the Benjamini-Hochberg threshold. In general, the power decreases with increase in heteroskedasticity at the QTL as well as the difference between the minor allele frequencies at the two loci. The powers are marginally higher for the chi-squares distribution compared to the normal distribution.

